# Using 3‐dimensional ultrasound islice technology for the diagnosis of developmental dysplasia of the hip

**DOI:** 10.1002/jum.15193

**Published:** 2019-12-09

**Authors:** Chenchen Geng, Hongtao Xu, Xinfeng Zhan, Li Li, Qian Song, Lu Zhang, Ling Ge

**Affiliations:** ^1^ Departments of Ultrasound Qilu Hospital of Shandong University Qingdao China; ^2^ Pediatric Orthopedics Qilu Hospital of Shandong University Qingdao China

**Keywords:** developmental dysplasia of the hip, reliability, 3‐dimensional ultrasound

## Abstract

**Objectives:**

This study aimed to investigate the reliability of 3‐dimensional (3D) ultrasound in screening for developmental dysplasia of the hip (DDH) by comparing the results with those of 2‐dimensional (2D) ultrasound.

**Methods:**

One hundred five infants who were younger than 6 months were enrolled in this study. All of the infants underwent 2D and 3D ultrasound scanning for DDH by novices and experts, and the images were graded by a lead expert. The scanning time and image grades were analyzed by Student *t* tests (*P* < .05). The consistency of the α angle measurement between the novices and experts was evaluated by the intraclass correlation coefficient (ICC).

**Results:**

The 105 infants included 34 boys and 71 girls. On 2D scanning, there was agreement between the experts about the correct diagnosis, whereas in the novice group, 41 infants had misdiagnoses. There were no misdiagnoses with 3D scanning in either group. In the novice group, the mean image grades ± SD were 4.2 ± 1.3 (2D ultrasound) and 8.1 ± 0.7 (3D ultrasound; *P* < .05). In the expert group, the mean image grades were 7.4 ± 1.0 (2D ultrasound) and 8.2 ± 1.0 (3D ultrasound; *P* < .05). There was no statistically significant difference between the groups in the grades for 3D ultrasound (*P* = .83). The scanning time for 3D ultrasound was shorter than that for 2D ultrasound in both groups (*P* < .05). In the novice group, the ICC of the α angle between the 2D and 3D ultrasound results was 0.34, and in the expert group, it was 0.92. The ICCs were 0.35 and 0.84, respectively when comparing 2D and 3D ultrasound results in the groups.

**Conclusions:**

Three‐dimensional ultrasound required less time and showed greater inter‐rater reliability than 2D ultrasound for detecting DDH.

**Abbreviations**
DDH: developmental dysplasia of the hip
ICC: intraclass correlation coefficient
3D: 3‐dimensional
2D: 2‐dimensional

Developmental dysplasia of the hip (DDH) is defined as an abnormal anatomic relationship between the femoral head and the acetabulum, referring to a wide spectrum of hip anomalies, from mild developmental abnormalities to frank dislocation. It is one of the most common musculoskeletal disorders in children and can cause claudication, hip pain, osteoarthritis, or even loss of function.^1^, ^2^, ^3^

Over the years, Barlow and Ortolani clinical maneuvers have been used by clinicians to assess the stability of hip; however, these physical examinations have limited sensitivity in the diagnosis of DDH in the early stages. Fortunately, DDH can be diagnosed earlier by ultrasound than by a physical examination.^4^, ^5^ The most popular method for hip screening is the Graf method with 2‐dimensional (2D) ultrasound; this method uses a standard image on the coronal position of the hip to measure the α and β angles. Both the quality of the image and the result depend on the operator's experience and proficiency.^6^ Considering the Graf method with 2D ultrasound, Jaremko et al^7^ noted that the repeatability between different operators was poor, and misdiagnosis occurred. Simon et al^8^ found that inexperienced examiners overdiagnosed DDH in patients more often than experienced examiners. Therefore, owing to the poor reliability and dependence on 2D ultrasound operators’ technical level, operators need professional training and experience to make an accurate diagnosis by 2D ultrasound.

With the development of new techniques, 3‐dimensional (3D) ultrasound has been used in many clinical settings for image acquisition, as it can show the 3D shape of the lesion and display the connections of each structure. Graf and Lercher^9^ applied 3D ultrasound to detect DDH for the first time; it had a much faster and simpler determination of a standard image than 2D ultrasound. Since then, several studies have aimed to detect DDH by 3D ultrasound.^6^, ^10^ The study by Jaremko et al^7^ also revealed that 3D ultrasound could be used to diagnose DDH. A comparison between 2D and 3D ultrasound in hip screening by Mostofi et al^11^ showed that 3D ultrasound was more reliable than 2D ultrasound. iSlice (Philips Healthcare, Amsterdam, the Netherlands) is a real‐time 3D analysis technology that can reconstruct the 3D volume and analyze a series of multiplanar images of objects after scanning, similar to computed tomography or magnetic resonance imaging.

With the development of mobile and 3D applications, ultrasound will be widely used in the future. In China, DDH occurs more often in infants who live in rural areas. Implementing universal scanning for DDH by inexperienced operators in rural clinics is a challenge. Hence, this study aimed to investigate the reliability of 3D ultrasound by comparing the time, repeatability, and image quality between 2D and 3D ultrasound examinations performed by novices and experts.

## Materials and Methods

### *Clinical Data*

This study was approved by the Ethics Commission of our hospital. From June 2018 to December 2018, 105 infants were enrolled in the study. The enrolled infants were younger than 6 months and healthy but with clinical symptoms such as asymmetry in the thigh folds, a positive clinical maneuver (Barlow or Ortolani) result, or apparent femoral shortening. All of the infants underwent 2D and 3D ultrasound screening of the hips by novices and experts successively. Before the examinations, the legal guardians of the infants were informed and signed an informed consent form.

### *Operators*

The operators included 2 groups: novices and experts. Five interns who had never performed hip ultrasound examinations were enrolled as the novices. Before the examination, 1 hour of training, including the acquisition of the standard plane and 3D image postprocessing, was conducted. Five professionals with 3 years of experience in hip ultrasound examinations were enrolled as the experts. After the examination, the quality of the images obtained by the groups was graded by a lead expert with 8 years of experience in hip ultrasound examinations. The lead expert reviewed the images acquired by the groups and made a diagnosis by selecting standard planes from 2D and 3D ultrasound results individually. The diagnosis made by the lead expert was considered the reference standard for the hip.

### *Images*

**Figure 1:**
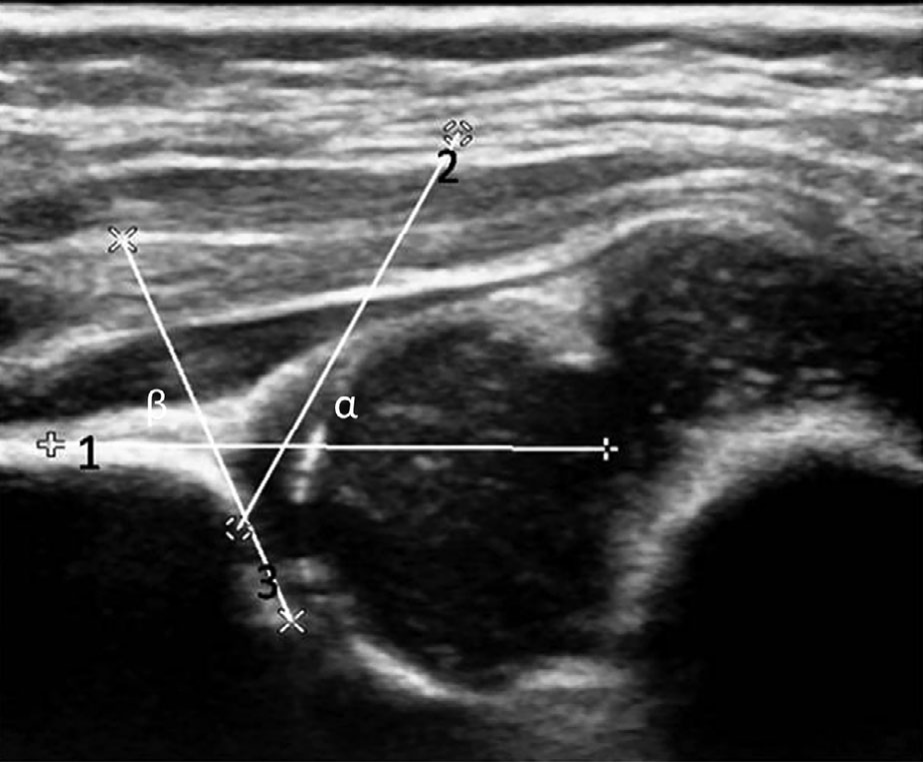
Measurement of the α and β angles of a normal hip. 1 indicates baseline; 2, bony acetabular line; and 3, cartilaginous acetabular line.

**Figure 2:**
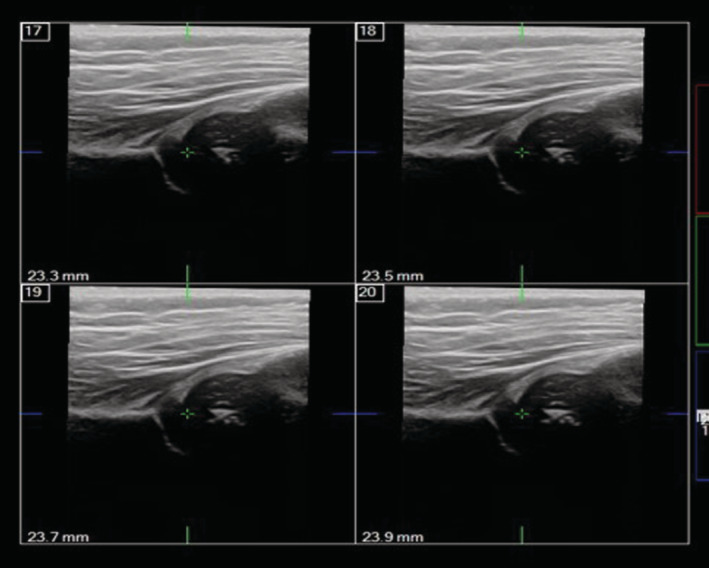
Example of iSlice 3D scans performed on the hip of an infant.

The infants were placed in the lateral position, and the lower limbs were naturally flexed. The novices and experts both scanned the hips with 2D and 3D ultrasound separately. Using Philips iU Elite platforms, a 9‐MHz linear (L9‐3) transducer for the 2D ultrasound and a 13‐MHz linear (13VL5) transducer for the 3D ultrasound were used for acquiring images. To obtain the 2D image, the transducer was placed on the greater trochanter perpendicular to the sagittal plane of the pelvis. The bony roof angle (α angle) was measured on the basis of the standard plane (Figure 1). Each hip was measured 3 times to obtain the mean value for further analysis. To obtain the 3D image, the transducer was placed on the greater trochanter and adjusted slightly to display the flat horizontal iliac wall. When iSlice started, the transducer was maneuvered over an angular range of ±15° for 3.2 seconds to produce ultrasound slices with a 0.2‐mm thickness (Figure 2). The operator selected a coronal slice that represented a standard Graf section to measure the α and β angles.

### *Grading the Image Quality*

All of the 2D and 3D images were graded by the lead expert, who was blinded to whether the image was obtained by a novice. On the image that was used for measuring the α and β angles, 10 landmarks (1 point for each: cartilage‐bone junction, visible full femoral head, inferior margin of the ilium, turning point of the ilium, flat horizontal iliac wall, acetabular roof, glenoid lip, articular capsule, synovial fold, and greater trochanter of the femur) were adopted, and each was graded.

### *Time*

The scanning time was the duration between the moment when the transducer was placed on the hip until the time the operator obtained satisfactory images, excluding preparation time and analysis time.

### *Statistical Analysis*

The statistical calculations were performed with SPSS version 19.0 software (IBM Corporation, Armonk, NY). Descriptive statistics were recorded as means ± standard deviations. The scanning time and image grades were analyzed by Student *t* tests (*P* < .05). The consistency of the measurement of the α angle between the novices and experts was evaluated by the intraclass correlation coefficient (ICC). As described in the literature, an ICC of 0 represented no agreement, and ICC values of greater than 0.6 were considered good agreement.

## Results

### *Basic Data*

**Figure 3:**
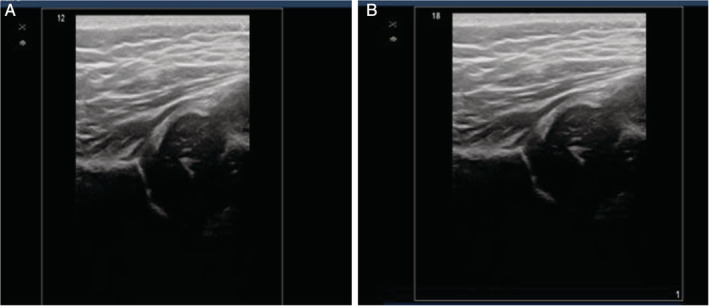
Example of 3D scans performed on the same hip. **A**, Performed by a novice operator. **B**, Performed by an expert operator.

The 105 infants included 34 boys and 71 girls. The infants were aged between 38 and 162 days. As standard planes could not be acquired because of the lack of a flat horizontal iliac wall in 5 infants in the novice group, they were excluded. One hundred infants (200 hips) were included in this study. The results included 142 hips with type I DDH, 33 hips with type IIa, 23 hips with type IIb, 1 hip with type IIc, and 1 hip with type D. On 2D scanning, all of the experts made correct diagnoses, whereas in the novice group, 8 hips with type I and 33 hips with type II were misdiagnosed. Neither group made any misdiagnoses with 3D ultrasound. In the novice group, the mean image grades for 2D and 3D ultrasound showed a statistically significant difference (*P* < .05; Table 1). In the expert group, the mean image grades for 2D and 3D ultrasound also showed a statistically significant difference (*P* < .05; Table 1). However, when comparing the image grades acquired by the groups for the 3D ultrasound, there was no statistically significant difference (*P* = .83; Figure 3). The scanning time for 3D ultrasound was also shorter than for 2D ultrasound in both groups, with a statistically significant difference (*P* < .05; Table 1).

**Table 1:** Results of the Novice and Expert Groups

	Novices		Experts	
Parameter	2D	3D	2D	3D
Time, s	30.2 ± 6.6	18.3 ± 4.9	23.7 ± 5.1	15.7 ± 3.9
Grade	4.2 ± 1.3	8.1 ± 0.7	7.4 ± 1.0	8.2 ± 1.0

### *Reliability*

**Figure 4:**
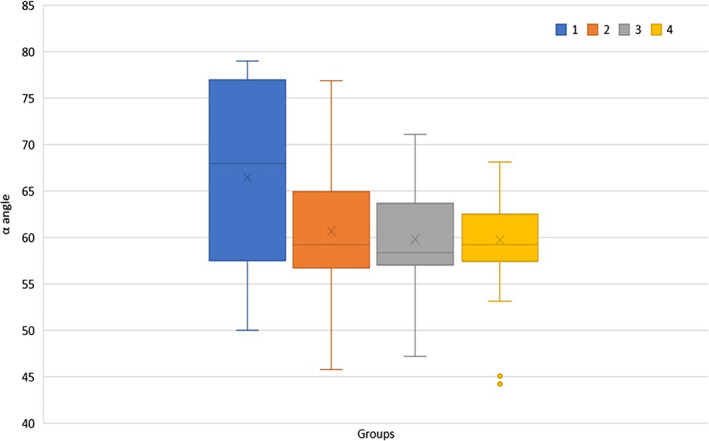
α angles of both groups. 1 indicates 2D by the novice group; 2, 3D by the novice group; 3, 2D by the expert group; and 4, 3D by the expert group.

We calculated the inter‐ and intra‐rater reliability between the novices and experts. The intra‐rater reliability, assessed by a 2‐way mixed‐effects models for consistency between 2D and 3D ultrasound in each group, was poor (ICC, 0.34) in the novice group but good (ICC, 0.92) in the expert group. The inter‐rater reliability was assessed by 2‐way random‐effects models between the groups, revealing poor reliability for 2D ultrasound (ICC, 0.35) but good reliability for 3D ultrasound (ICC, 0.84; Figure 4).

## Discussion

Developmental dysplasia of the hip refers to a spectrum of hip abnormalities, from dysplasia to subluxation or even dislocation,^10^ with an incidence of 0.1% to 2.0% in surviving neonates.^11^ Early diagnosis and treatment can effectively restore the hip and improve the prognosis in infants younger than 6 months, but it requires early detection in the suspected hip.^12^, ^13^ Ultrasound is an excellent tool for assessing hips, as it has the advantages of no radiation, easy repeatability, and a relatively low cost. Physicians use the Graf method by conventionally measuring the α and β angles on 2D ultrasound images. To determine the application of 3D ultrasound in hip examinations, we searched the PubMed database using the key words “3D,” “ultrasound,” and “hip” in the Abstract/Title field, with dates from 1996 to 2018. We found that some researchers had been using the 3D ultrasound method for hip scanning, but with a different focus, since 1996. In the study by Graf and Lercher^9^ in 1996, the results suggested that determining the standard plane was quicker and simpler with 3D ultrasound than with 2D ultrasound. Because of technological restrictions, the volume box system used could not acquire multiplanar images of the hip simultaneously.^9^ Hünerbein et al^10^ investigated the feasibility and applications of 3D ultrasound in the evaluation of multiple musculoskeletal disorders, including DDH. Jaremko et al^7^ revealed that 3D ultrasound displayed the full acetabular shape and improved DDH assessment accuracy, as the measured α angles were not affected by the transducer position as they were in 2D scans. The study performed by Mostofi et al^11^ revealed that 3D ultrasound had greater reliability and feasibility than 2D ultrasound by comparing the image qualities. Mabee et al^14^ found that 3D ultrasound could capture the entire acetabular shape and reproducible standard central plane when considering different observers.

Scholars inferred that 3D ultrasound had advantages over 2D ultrasound because it included multiplanar visualization, reduced the dependence on the operator, and achieved standardization of measurements. Compared to previous studies, we had additional foci, including time, repeatability, and image quality, in this study to compare images acquired by novices and experts using 2D and 3D ultrasound. Considering the multiplanar images of the hip obtained by iSlice technology, we believe that iSlice has 3 main advantages: namely, it is less dependent on operator experience; it is easier to master; and it produces better image quality than 2D ultrasound.

In the Graf method, only a single standard image including landmarks such as the greater trochanter, cartilage‐bone junction, glenoid lip, and flat horizontal iliac wall represents the hip; thus, the operator's technical ability and experience are very important. It is difficult for novices to master the Graf method with 2D ultrasound because the operator needs to adjust the transducer constantly to obtain a standard image. In this study, the mean time for image acquisition in the novice group was 28.8 ± 5.6 seconds, which was much longer than that in the expert group, as it was difficult to obtain the standard image before the infant began crying, owing to their discomfort. Because only a static image is obtained, if it is substandard, the infant needs to be examined again. Additionally, we could not determine whether the lowest point of the inferior margin of the ilium was visible on the static image. Total ultrasound exposure should be kept as low as reasonably achievable while optimizing diagnostic information.^15^ Using iSlice technology, the operator placed the transducer on the greater trochanter and started scanning until the flat horizontal iliac wall emerged. Then, 3 perpendicular reconstructed planar sections, including the sagittal, transverse, and coronal planes, from the posterolateral to anteromedial greater trochanter were obtained. A complete 3D rendering of the hip is conducive to repeated analyses, remote consultation, and quality control. As previously described, 3D ultrasound saves much more time and is easier to operate than 2D ultrasound. In particular, the infant should not move in the process of scanning, as once the hip moves, the 3D volume will be vague; this is why 5 infants were excluded in our study.

Poor image quality may lead to misdiagnosis; the main reason for misdiagnosis in this study was the lack of a flat horizontal iliac wall on the plane. In the novice group, 41 cases were misdiagnosed with 2D ultrasound. Moreover, 8 normal hips were diagnosed as type II. If the results were not corrected by the lead expert, the false‐positive results would have led to unnecessary treatment and increased anxiety in the families. For the other 33 type II hips that were misclassified as normal, false‐negative results would have caused delayed treatment and a poor prognosis. With iSlice technology, it is easy to find a more‐or‐less standard plane for measuring the 3D volume data. With 3D ultrasound, the novices produced no false‐positive or false‐negative results, suggesting that 3D ultrasound is more reliable than 2D ultrasound.^11^, ^16^

This study had certain limitations. First, we had no external comparison standard, except ultrasound, as radiography and magnetic resonance imaging are not used to examine hips in those who are younger than 6 months. Second, we had no long‐term follow‐up data to verify the clinical and ultrasound results.

In conclusion, 3D ultrasound required less time and showed greater inter‐rater reliability than 2D ultrasound for detecting DDH. Three‐dimensional ultrasound can reduce the technical dependence on the operator and increase the ease of operation, resulting in its increasing use.
